# Diagnostic, Prognostic, and Predictive Role of Ki67 Proliferative Index in Neuroendocrine and Endocrine Neoplasms: Past, Present, and Future

**DOI:** 10.1007/s12022-023-09755-3

**Published:** 2023-02-17

**Authors:** Stefano La Rosa

**Affiliations:** 1 Unit of Pathology, Department of Medicine and Surgery, University of Insubria, Via O. Rossi 9, Varese, 21100 Italy; 2 Unit of Pathology, Department of Oncology, ASST Sette Laghi, Varese, Italy

**Keywords:** Ki67, Neuroendocrine neoplasms, Endocrine neoplasms, Prognosis, Classification

## Abstract

The introduction of Ki67 immunohistochemistry in the work-up of neuroendocrine neoplasms (NENs) has opened a new approach for their diagnosis and prognostic evaluation. Since the first demonstration of the prognostic role of Ki67 proliferative index in pancreatic NENs in 1996, several studies have been performed to explore its prognostic, diagnostic, and predictive role in other neuroendocrine and endocrine neoplasms. A large amount of information is now available and published results globally indicate that Ki67 proliferative index is useful to this scope, although some differences exist in relation to tumor site and type. In gut and pancreatic NENs, the Ki67 proliferative index has a well-documented and accepted diagnostic and prognostic role and its evaluation is mandatory in their diagnostic work-up. In the lung, the Ki67 index is recommended for the diagnosis of NENs on biopsy specimens, but its diagnostic role in surgical specimens still remains to be officially accepted, although its prognostic role is now well documented. In other organs, such as the pituitary, parathyroid, thyroid (follicular cell-derived neoplasms), and adrenal medulla, the Ki67 index does not play a diagnostic role and its prognostic value still remains a controversial issue. In medullary thyroid carcinoma, the Ki67 labelling index is used to define the tumor grade together with other morphological parameters, while in the adrenal cortical carcinoma, it is useful to select patients to treated with mitotane therapy. In the present review, the most important information on the diagnostic, prognostic, and predictive role of Ki67 proliferative index is presented discussing the current knowledge. In addition, technical issues related to the evaluation of Ki67 proliferative index and the future perspectives of the application of Ki67 immunostaining in endocrine and neuroendocrine neoplasms is discussed.

## Introduction

The Ki67 antibody was created in 1983 by immunizing mice with nuclei of the Hodgkin lymphoma cell line L428. It was called “Ki” referring to the city Kiel where the research team worked and “67” referring to the original clone in the 96-well plate [1]. Although the function of the identified protein was not known, it was immediately clear that it was involved in cell proliferation being detected in proliferating normal and neoplastic cells. Unfortunately, the original Ki67 antibody only worked on frozen tissues and for this reason its use in routine practice was limited. Some years later, the monoclonal antibody MIB1, which works in formalin-fixed and paraffin-embedded tissues, was generated and since then the use of Ki67 immunohistochemistry in routine practice has become easy [2].

The prognostic role of Ki67 proliferative index in neuroendocrine tumors (NETs) was identified for the first time in 1996 by two independent Italian research teams [3, 4], and since then, many studies have been performed to evaluate its prognostic, diagnostic, and predictive role in other digestive neuroendocrine neoplasms (NENs). The encouraging results obtained in these investigations played a motor role to study the biological role of Ki67 in NENs located in other organs (i.e., pituitary, parathyroid, lung, etc.), but also in endocrine tumors such as follicular cell-derived thyroid tumors and adrenal cortical neoplasms. A large amount of information is now available and published results globally indicate that Ki67 proliferative index is a useful prognostic, diagnostic, and predictive biomarker, although some differences exist in relation to tumor site and type.

In the present review, the most important information on this topic will be presented and discussed. The current knowledge and the future perspectives of the application of Ki67 immunostaining will be analyzed in endocrine and neuroendocrine neoplasms arising in different organs and systems.

## Ki67 Proliferative Index in Different Endocrine and Neuroendocrine Neoplasms

### Pituitary Neuroendocrine Tumors (PitNETs)

**Fig. 1:**
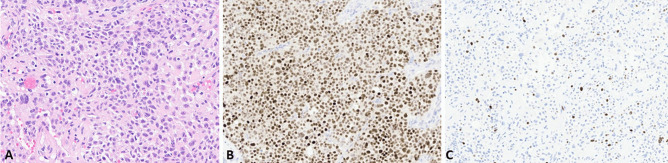
Silent corticotroph PitNET (**A**) positive for TPIT (**B**) with a Ki67 index > 3% (**C**)

Anterior pituitary tumors represent a peculiar well-differentiated neuroendocrine tumor type characterized by specific features depending on its site of origin and include distinct entities producing different hormones with variable propensity to local infiltration and/or metastatic dissemination [5]. For this reason, they are no longer considered “benign” tumors as in the past and, consequently, the terminology for their definition has recently changed from “pituitary adenomas” to “pituitary neuroendocrine tumors (PitNETs),” to better cover the spectrum of different biological characteristics [5, 6]. Once that this conceptual change has been made, the pathologist’s goal in the diagnostic work-up of PitNET is the identification of parameters able to recognize those cases associated with significant risk of recurrence or local infiltration. Considering the proposal of a common classification framework to unify the nomenclature of neuroendocrine neoplasms (NENs) arising in different organs, which is based on morphological differentiation and proliferation [7], the role of Ki67 proliferative index may be important to better define PitNETs and to stratify them in different prognostic categories. Although several attempts have been made to explore it, the predictive value of Ki67 proliferative index in PitNETs still remains to be definitively confirmed [8, 9]. The prognostic role of Ki67 index has been explored and its integration with morphological and radiological evidence of local infiltration seemed promising for identifying tumors at high risk of local recurrence and/or infiltration [10]. This approach has been confirmed in different series, which also demonstrated the strong prognostic role of PitNET subtype. Indeed, certain tumor types behave per se in a more aggressive fashion such as immature PIT1-lineage tumors, Crooke cell tumors, null cell tumors, silent corticotroph tumors, and sparsely granulated somatotroph and corticotroph tumors [11–14]. In a recent paper, an integrated multiparametric approach demonstrated that tumor type (mainly ACTH subtype), Ki67 index ≥ 3% (Fig. 1), mitotic count > 2/10 HPF, local invasion, “grade 2” of the Trouillas’ grading, and p53 protein expression were predictor of disease recurrence or progression [15]. Taken together, all these findings suggest that the Ki67 proliferative index, although may give an indication on PitNETs biology, does not have alone an independent prognostic role and needs to be integrated with other parameters. Tumor subtype currently has a major prognostic meaning [16] reflecting what observed in other NETs such as gastric ECL-cell NETs for which the most important prognostic marker is tumor subtype rather than the proliferation index alone [17].

### Head and Neck Neuroendocrine Neoplasms

The new 2022 classification of head and neck tumors [18] includes a specific chapter on the classification of NENs and represents an important evolution respect to the previous classification, which used a confounding terminology that has created uncertainties among pathologists and clinicians [19]. The new classification is in line with the common classification framework proposed by WHO/IARC [7] and separates head and neck NENs into well differentiated neuroendocrine tumors (NETs) and poorly differentiated neuroendocrine carcinomas (NECs), although, for obscure reasons, it does not include mixed neuroendocrine/non-neuroendocrine neoplasm (MiNEN) as a specific entity [18]. In this context, Ki67 has been introduced as parameter for the classification, not alone but integrated with other morphological parameters such as the presence of necrosis and mitotic count, reflecting the morphological approach used in the lung (see below). The combination of Ki67 index, mitotic count, and presence of necrosis allows to classify tumors into NET G1, NET G2, and NET G3, the last still considered as a provisional entity due to limited data available in the literature (Table 1). In the head and neck region, the distinction between NET G1 and NET G2 does not use the same criteria of the digestive system. Indeed, both entities are characterized by a Ki67 index < 20% [20, 21] and their separation is based on morphological criteria using necrosis and mitoses. Obviously, this Ki67 threshold is too high and does not help the distinction between G1 and G2 NETs; thus, a more specific cutoff for their separation is needed [22] and will be welcome. Conversely, Ki67 count is crucial to identify those NETs with higher biological aggressiveness and characterized by a Ki67 percentage higher than 20% that are defined NET G3. Since the Ki67 value (> 20%) is the same observed in NECs, the differential diagnosis between NET G3 and NECs, either small or large cell subtype, is mainly based on morphology, associated with molecular profile, which can partly be evaluated using immunohistochemical surrogate-marker analyses.

**Table 1:** The 2022 WHO classification of neuroendocrine neoplasms of the head and neck. Modified from [18]

Tumor category	Morphological differentiation	Diagnostic criteria
Neuroendocrine tumor, grade 1 (NET G1)	Well differentiated	-No necrosis -Mitotic count: < 2 mitoses per 2 mm^2^ -Ki67 index: < 20%
Neuroendocrine tumor, grade 2 (NET G2)	Well differentiated	-Necrosis and/or 2–10 mitoses per 2 mm^2^ -Ki67 index: < 20%
Neuroendocrine tumor, grade 3 (NET G3)*	Well differentiated	-Mitotic count: > 10 mitoses per 2 mm^2^ -Ki67 index: > 20%
Small cell neuroendocrine carcinoma (SCNEC)	Poorly differentiated, small cell morphology	- > 10 mitoses per 2 mm^2^ -Ki67 > 20% (often > 70%)
Large cell neuroendocrine carcinoma (LCNEC)	Poorly differentiated, large cell morphology	- > 10 mitoses per 2 mm^2^ -Ki67 > 20% (often > 50%)

^*^Provisional entity

**Fig. 2:**
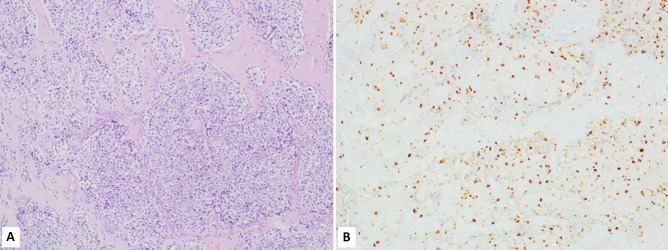
Olfactory neuroblastoma showing the typical lobular architecture (**A**) and presenting a Ki67 proliferative index > 20% (**B**), which is associated with a worse prognosis than observed in cases with Ki67 < 20%

Olfactory neuroblastoma is a peculiar nonepithelial neoplasm showing neuroendocrine differentiation and including different clinically relevant subgroups mainly defined using a morphological grade (Hyams grade) [18]. However, recent studies have demonstrated that the Ki67 labelling index has a prognostic relevance and, in particular, Ki67 index > 20% (Fig. 2) is associated with a worse prognosis [23, 24].

In conclusion, the 5th edition of the WHO classification of head and neck tumors represents an important evolution respect the previous one and introduces, for the first time, a diagnostic role of Ki67 labelling index that, however, needs to be better characterized in terms of refined cut-offs to use to precisely define the different entities.

### Thyroid Tumors

#### Follicular Cell-Derived Neoplasms

The diagnostic role of Ki67 proliferative index in follicular cell-derived neoplasms has been investigated in several papers. In follicular and oncocytic neoplasms, the Ki67 index has been found to be lower in adenomas than in carcinomas [25–28], although overlaps exist between the two entities [29–32]. More recently, the Ki67 proliferative cutoff of 4% has been proposed to separate these two entities, suggesting a promising role of Ki67 in the diagnostic work-up that, however, needs to be validated [33]. For this reason, the differential diagnosis between adenoma and carcinoma, either follicular or oncocytic, still resides on morphology evaluating capsular and vascular invasion.

Ki67 index has also been investigated as a possible prognostic marker in the group of minimally invasive follicular carcinoma. Although it was found to correlate with disease-free survival, its impact was less powerful than vascular invasion and patient’s age [34]. The prognostic role of Ki67 index in widely invasive follicular carcinoma has been explored as well. Although Ki67 index > 5% seems associated with high risk of recurrence, there are not sufficient data supporting its powerful when compared with other markers such as vascular invasion [35].

Additional efforts have been made to explore the possible role of Ki67 in identifying aggressive follicular cell-derived carcinomas (i.e., poorly differentiated thyroid carcinoma -PDTC-) and in stratifying patients in different prognostic categories. Several papers suggested that the increase of Ki67 proliferative index parallels the decrease in differentiation and that PDTC generally shows a 10–30% Ki67 index [25]. However, an optimal diagnostic cut-off able to identify PDTC still remains to be defined. For this reason, in the 5th edition of WHO classification of endocrine tumors, the group of high grade follicular-derived carcinomas, which includes PDTC and differentiated high-grade thyroid carcinoma (DHGTC), is defined only using morphological parameters such mitotic count and the presence of necrosis [6].

The diagnosis of papillary thyroid carcinoma (PTC) resides on nuclear morphology and Ki67 does not have any diagnostic role in this context. However, Ki67 index has been explored as a potential prognostic marker. Some studies have suggested a possible prognostic role when combined with other biomarkers such as CK19 expression or *TERT* promoter/*BRAF*^*V600E*^ mutations [36–38], but, in principle, aggressive histological variants of PTC generally show higher Ki67 labelling index than classical variant [25], although some exceptions have been reported.

The formerly known cribriform-morular variant of PTC [39] is now redefined as cribriform-morular thyroid carcinoma since this tumor is considered as a distinct malignant thyroid neoplasm of uncertain histogenesis [40] with relatively indolent behavior but relatively high Ki67 index [39].

In conclusion, all these findings suggest that Ki67 proliferative index does not have a prominent value in thyroid pathology. The currently used well-defined morphological diagnostic algorithms allow the correct and specific diagnosis of thyroid neoplasms, which also correlates with prognosis. The prognostic role of Ki67 index is promising but need to be better defined. In addition, it is worth noting that no well standardized methods for Ki67 count have been validated in the thyroid. Consequently, published data that have been obtained using different protocols proposed different Ki67 cut-off values making the results hard to compare [41–45].

#### Medullary Thyroid Carcinoma

**Fig. 3:**
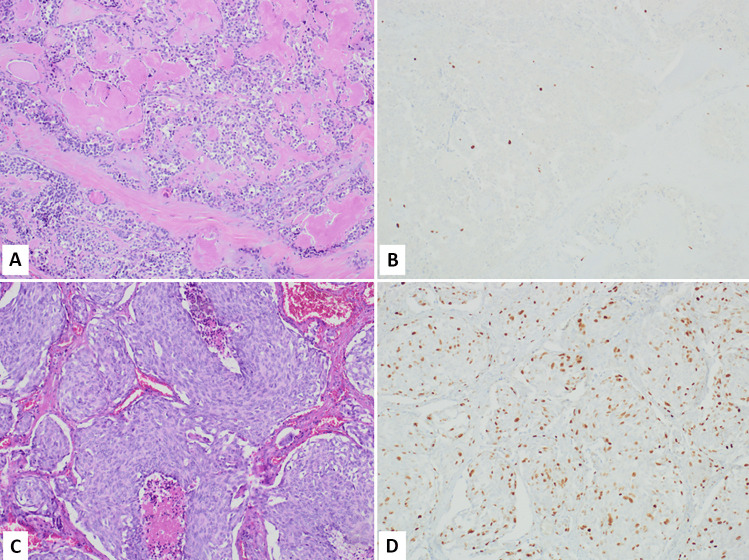
Low-grade medullary thyroid carcinoma (**A**) showing a Ki67 proliferative index < 5% (**B**). High-grade medullary thyroid carcinoma showing necrosis (**C**) and a Ki67 proliferative index > 20% (**D**)

Medullary thyroid carcinoma (MTC) is a primary neuroendocrine neoplasm of the thyroid composed of calcitonin-secreting C-cells. It is a rare but relatively aggressive cancer and can be sporadic or can arise in the setting of hereditary diseases [6]. In hereditary MTC early detection of cancer, performed using biochemical and/or genetic screening, plays a major role in improving patient’s outcome [46, 47]. The prediction of survival in patients with sporadic MTC additionally includes other prognostic markers that negatively influence the survival, such as large tumor size, extrathyroidal extension, old age, male gender, serum calcitonin and CEA levels, and *RET* mutations in exons 15 and 16 [46–50]. With the aim to improve the stratification of patients in different prognostic categories, two grading systems based on proliferation (mitotic count and/or Ki67 index) and necrosis have been proposed: the Sydney grading system [51] and the Memorial Sloan Kettering Cancer Center grading system (MSKCC) [52]. Starting from these observations, a group of internationally recognized experts in thyroid pathology from the USA, Europe, and Australia recently proposed the International Medullary Thyroid Carcinoma Grading System (IMTCGS) that, combining proliferation (mitotic count and Ki67 proliferative index) and necrosis, separates MTC in two different prognostic categories: low and high grade MTC (Fig. 3 and Table 2) [53]. The IMTCGS system has been validated in other series [54] and appears well reproducible [55]. In conclusion, recent findings have demonstrated that Ki67 labelling index plays a prognostic role in MTC, although the better prognostic stratification of patients is obtained combining Ki67 index with necrosis (IMTCGS system).

**Table 2:** International Medullary Thyroid Carcinoma Grading System (IMTCGS) [53]

Medullary thyroid carcinoma (MTC)	Diagnostic criteria
Low grade	No necrosis and mitoses < 5 per 2 mm^2^ and Ki67 < 5%
High grade	Necrosis and/or mitoses ≥ 5 per 2 mm^2^ and/or Ki67 ≥ 5%

### Parathyroid Neoplasms

The differential diagnosis between parathyroid adenoma and carcinoma depends on the presence of at least one of the following morphological features: angioinvasion, lymphatic invasion, perineural invasion, invasion into the adjacent structures/organs, or, obviously, the presence of regional or distant metastasis [6, 56]. However, since their distinction is not rarely problematic [57], several biomarkers have also been investigated during the last years to help in solving this issue. Among them, Ki67 index was evaluated, and the first studies were published in the last decade of the twentieth century [58]. Ki67 index was found to be higher in parathyroid tumors and hyperplasia than in normal glands [59] and, among tumors, in carcinoma than in adenomas [60]. Ki67 index higher than 5% has been suggested to be associated with malignancy [61], but due to the overlap of Ki67% values between adenoma and carcinoma and to the observation that in secondary parathyroid hyperplasia and multiglandular parathyroid disease Ki67 index may be higher than in adenoma and carcinoma [59], this marker cannot be used alone [62]. A diagnostic nomogram including five biomarkers (parafibromin, Rb, Ki67, PGP9.5, and galectin 3) has been proposed but Ki67 did not show as good performance as the staining for parafibromin in distinguishing adenoma from carcinoma [63]. In addition to its diagnostic role, Ki67 index has also been explored as a prognostic marker in parathyroid tumors, without definitive results [64, 65]. For these reasons, Ki67 it is not currently included in the diagnostic algorithm of parathyroid neoplasm for the differential diagnosis between parathyroid adenoma and carcinoma, although it may give an overview on the proliferative activity of the tumor. However, Ki67 should be evaluated as a complementary possible prognostic marker and reported as a continuous variable since no definite prognostic cutoff has been identified yet.

### Thoracic Neuroendocrine Neoplasms

The role of Ki67 proliferative index in the diagnosis and prognostic evaluation of lung neuroendocrine tumors (carcinoids) has been matter of debate in recent years. Its more and more emerging diagnostic and prognostic relevance in the work-up of digestive neuroendocrine neoplasms has supported its application in the diagnosis of lung NENs and several papers have been published in recent years on this topic [66]. However, its introduction as a routine diagnostic marker for lung NENs has encountered some difficulties. The major obstacle has resided in the fact that morphological criteria (mitotic count and necrosis) traditionally used for diagnosing lung NENs in surgical specimens works well in identifying different NEN entities characterized by different morphological, molecular and prognostic features: typical carcinoid (TC), atypical carcinoid (AC), large cell neuroendocrine carcinoma (LCNEC), and small cell carcinoma (SCLC). Consequently, the Ki67 index has been considered not adding additional and practical advantage in this setting.

**Fig. 4:**
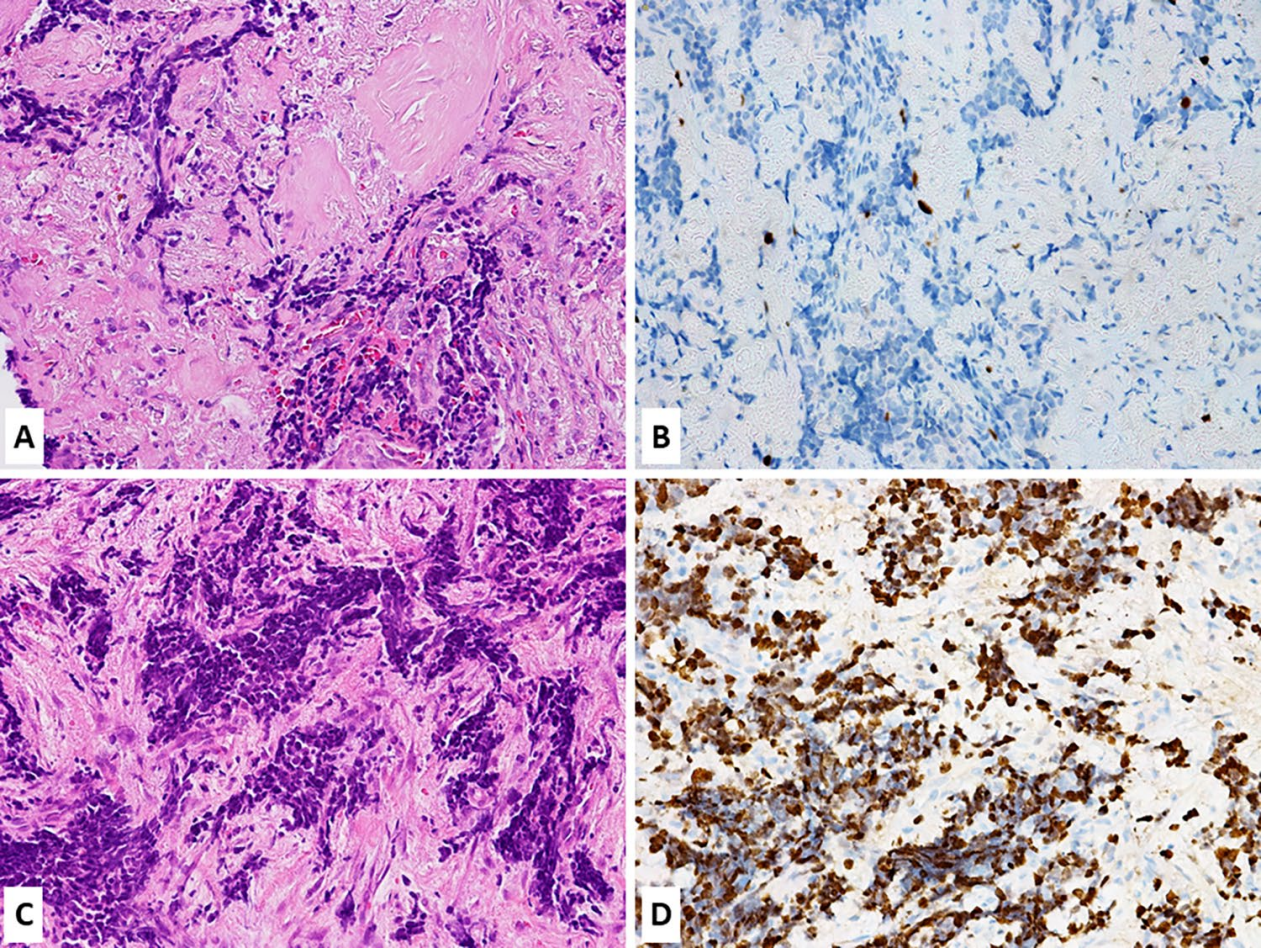
Lung biopsies of neuroendocrine neoplasms frequently present crush artifacts creating difficulties in interpreting the morphological picture. In this context, Ki67 is useful to distinguish NET (carcinoid) from NEC. **A** refers to a NET, which shows low Ki67 labelling (**B**). (**C)** is a small cell carcinoma presenting high Ki67 immunolabelling (**D**) (courtesy of Prof. Giuseppe Pelosi, University of Milan, Milan, Italy)

However, Ki67 has emerged as a potentially useful marker in the diagnostic work-up of lung NENs in biopsy specimens [67]. Indeed, extensive crush artifacts are frequently observed in tissue fragments obtained with biopsy procedures and this creates difficulties in interpreting the morphological picture and to get the right pre-operatory diagnosis, which is strategic for patients’ management. In this context, the Ki67 labeling index is helpful because lung NETs (carcinoids) show lower Ki67 rates (< 20–30%) than lung NECs, both small and large cell subtypes, where the Ki67 index is over 50% and not rarely exceed 70–80% [67–69]. For this reason, Ki67 immunohistochemistry is strongly recommended in the diagnostic work-up of bronchial or lung biopsies (Fig. 4) when the neuroendocrine nature of the lesion is confirmed by the immunoreactivity for neuroendocrine markers (synaptophysin, chromogranin, and INSM1).

As above discussed, the Ki67 index is not currently essential for the diagnosis and classification of lung NENs, which is based on morphological features [70]. However, Ki67 has been introduced in the last 2021 WHO classification (5th Edition) as a parameter helping the distinction among different entities, with a range of percentages of Ki67 positive cells indicated for each entity: up to 5% for typical carcinoid, up to 30% for atypical carcinoid, and > 30% for NECs (either small or large cell subtypes) [70]. Interestingly, a growing burden of information has indicated that the Ki67 labelling index also plays a prognostic role in lung NENs and is useful for better defining the biological behavior of different lung NEN subtypes and, especially, of NETs (carcinoids) [69, 71, 72]. Reflecting the classification used for digestive NENs (see below) and considering the recent proposal for a common classification framework of NENs of different sites [7], a three-tiered stratification may also be proposed with TCs corresponding to G1 NETs, ACs to G2 or G3 NETs and LCNEC and SCNECs to NECs. However, while in the digestive system, the cutoffs to separate G1 from G2 and G3 categories have been accepted, in the lung they are not well defined, and especially they do not have a direct correspondence with the specific morphologically defined entities. In other words, there are not universally accepted established Ki67 cutoff points able to separate TC from AC and for distinguishing carcinoids from both small and large cell NECs [69, 72]. However, based on expert opinion, it has been suggested that tumors with a Ki67 < 5% likely correspond to TCs (NET G1), those with Ki67 > 5% to ACs (NET G2) and those with Ki67 index > 30% likely correspond to NECs [66].

**Fig. 5:**
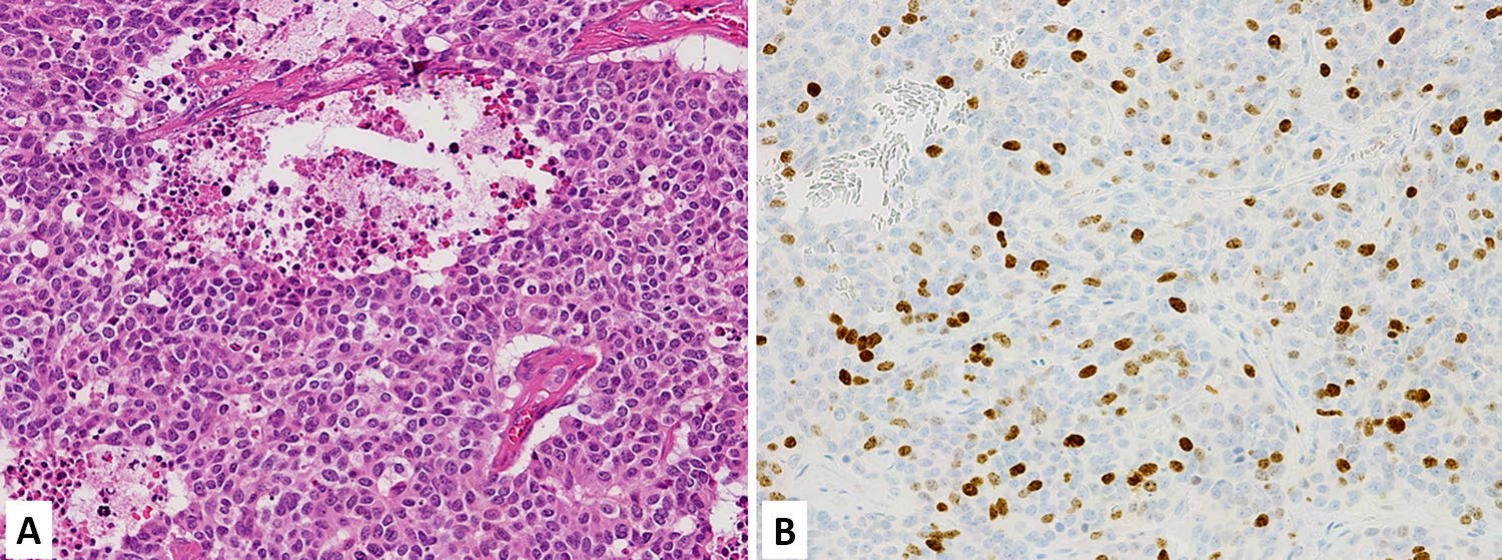
Lung NET G3 (atypical carcinoid with high proliferative rate). The tumor is composed of well-differentiated cells and a punctate necrosis is visible (**A**). The Ki67 proliferative index is > 30% (**B**) (courtesy of Prof. Giuseppe Pelosi, University of Milan, Milan, Italy)

Another recent and clinically relevant issue regarding the role of Ki67 in the diagnosis and prognosis of lung NETs (carcinoids) is the identification of a subset of lung NETs (ACs) showing high proliferative rates (Fig. 5) [73–76]. These cases associate a well differentiated morphology with a high proliferation rate (> 10 mitoses per 2 mm^2^ and > 30% Ki67 index) and considering the last parameter they should be classified as LCNECs. Interestingly, these tumors show a molecular profile closer to carcinoids (NETs) rather than to NECs, lacking *RB1* or *TP53* mutations and showing a low total mutation burden, and/or presence of MEN1 mutations [77]. In addition, these tumors show a prognosis different from that of conventional LCNEC. For these reasons, the term “carcinoid tumors with elevated mitotic counts and/or Ki67 proliferation rates” has been proposed in the 5th edition of WHO classification [70]. These NETs [78] likely correspond to NET G3 well described and characterized in the digestive system [79], underlining once again a sort of parallelism between digestive and lung NENs and consequently supporting the use of a common classification framework recently proposed by WHO/IARC [7].

In conclusion, although the diagnosis and the management of patients with lung NENs currently rely on the histologic classification, the growing relevance of Ki67 as diagnostic and prognostic marker has implied the introduction of the evaluation of Ki67 index in at least three setting of the diagnostic pathology work-up. First, it is now mandatory to perform Ki67 immunohistochemistry in biopsy specimens to avoid overdiagnosing NETs (carcinoids) as NECs. Second, to identify those TCs (with > 5%) that may behave more aggressively than expected or, conversely, those ACs (with Ki67 < 5%) that may show a less aggressive fashion than expected [80–84]. Third, to identify those ACs with high proliferation (likely corresponding to the NET G3 category accepted in the digestive system) that have been traditionally considered LCNECs but that show a prognostic feature in the middle between NET and NEC.

The personal opinion of the author is that, not forgetting some specific site-related peculiarities, the classification of lung NENs should be uniformed with that of the digestive system including lung NET G1, NET G2, NET G3 as well-differentiated tumors and small and large cell NECs as poorly differentiated ones (Table 3). The term carcinoid should be abandoned also for clinical reasons because the carcinoid syndrome, except for rare cases of atypical carcinoid syndrome, is not observed in lung NETs and consequently this fascinating but old term may be confounding [85].

**Table 3:** Proposed classification of lung neuroendocrine neoplasms. Modified from [7]

Proposed terminology	Current terminology	Morphological differentiation	Diagnostic criteria
Neuroendocrine tumor, grade 1 (NET G1)	Typical carcinoid	Well differentiated	-No necrosis - < 2 mitoses per 2 mm^2^ -Ki67 index: < 5%
Neuroendocrine tumor, grade 2 (NET G2)	Atypical carcinoid	Well differentiated	-Necrosis and/or 2–10 mitoses per 2 mm^2^ -Ki67 index: 5–30%
Neuroendocrine tumor, grade 3 (NET G3)	Carcinoid with elevated mitotic counts and/or Ki67 proliferation index	Well differentiated	-Mitotic count: > 10 mitoses per 2 mm^2^ -Ki67 index: > 30%
Small cell neuroendocrine carcinoma (SCNEC)	Small cell carcinoma	Poorly differentiated, small cell morphology	- > 10 mitoses per 2 mm^2^ -Ki67 index: 30–100%
Large cell neuroendocrine carcinoma (LCNEC)	Large cell neuroendocrine carcinoma	Poorly differentiated, large cell morphology	- > 10 mitoses per 2 mm^2^ -Ki67 index: 30–100%

Thymic NENs are currently classified and defined using the morphological criteria used in the lung [70]. They are rare accounting for about 0.4% of all NENs of the body and, for this reason, there are not published studies including large series. In this context, the available data on the diagnostic and prognostic role of Ki67 proliferative index are limited [86, 87]. From a diagnostic point of view, Ki67 is suggested as desirable diagnostic criterium for the differential diagnosis between carcinoid (NET) and NEC (both large and small cell) in biopsy specimens, as proposed in the lung [70]. The prognostic role of Ki67 in thymic NETs has been poorly investigated and finding on small series seems to suggest that the cutoff of 10% is able to separate patients into two different groups [87].

### Gut and Pancreatic Neuroendocrine Tumors

**Fig. 6:**
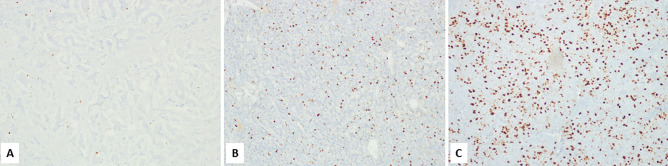
The Ki67 proliferative index is used to grade digestive neuroendocrine tumors (NETs). NET G1 is characterized by Ki67 index < 3% (**A**), NET G2 by Ki67 index > 3% but < 20% (**B**), while NET G3 shows a Ki67 index > 20% (**C**, courtesy of Prof. Silvia Uccella, Humanitas University, Milan, Italy)

Most of published studies on Ki67 proliferative index have been performed in gut and pancreatic NENs, probably because the first investigations on the prognostic role of Ki67 index were performed in the pancreas [3, 4]. Since these first pioneer studies, Ki67 index has become increasingly important in the pathology work-up of NENs reaching a crucial pivotal role in 2006 when the European Neuroendocrine Tumor Society (ENETS) proposed a 3-tiered grading system with prognostic relevance [88, 89]. The utility of this grading system has successively been validated in several studies [90] and, for this reason, was accepted by the WHO/IARC in 2010 [91]. With only minor changes respect to the original ENETS proposal, this grading system is currently in use (Fig. 6 and Table 4) [79, 92]. However, although, as a whole, the Ki67 proliferative index is able to give prognostic information, some subtle but important differences related to tumor type and site of origin need to be considered in routine practice.

**Table 4:** Classification and proliferative grade of gastrointestinal and pancreatic neuroendocrine neoplasms. Modified from [79]

Tumor type	Morphological differentiation	Mitotic count (mitoses/2 mm^2^)	Ki67 index
Neuroendocrine tumor, grade 1 (NET G1)	Well differentiated	< 2	< 3%
Neuroendocrine tumor, grade 2 (NET G2)		2–20	3–20%
Neuroendocrine tumor, grade 3 (NET G3)		> 20	> 20%
Neuroendocrine carcinoma, small cell type (SCNEC)	Poorly differentiated	> 20	> 20%
Neuroendocrine carcinoma, large cell type (LCNEC)		> 20	> 20%
Mixed neuroendocrine/non-neuroendocrine neoplasm (MiNEN)	Well or poorly differentiated	Variable	Variable

#### Stomach

The majority of gastric NETs are histamine-producing ECL-cell NETs that include at least five subtypes characterized by different clinico-pathologic features, strongly related per se to prognosis. Indeed, NETs not associated with hypergastrinemia (type 3) behave worse than NETs associated with high gastrin serum levels (type 1, type 2, type 4, and type 5) that, in turn, have different prognostic profile depending on the clinico-pathologic background [93]. In this context, the prognostic role of the Ki67 proliferative index needs to be integrated with the intrinsic biological characteristics of different NET types. In type 1 ECL-cell NETs (associated with chronic atrophic gastritis), Ki67 index does not play a major role since G1 and G2 tumors show similar behavior [17]. Conversely, in type 3 NET (not associated with hypergastrinemia and arising in normal gastric mucosa), Ki67-based grading stratifies patients in different prognostic groups [17]. Published data on the Ki67 role in type 2 NETs (MEN1 patients with gastrinoma) and type 4 ECL-cell NETs (associated with defect/lack of proton pump function) are not sufficient to have a clinical application. Type 5 ECL-cell NET, observed in patients long term treated with proton pump inhibitors and arising in normal or slightly hyperplastic peri-tumoral oxyntic mucosa (oxyntic gland dilatation, parietal cells with apocrine-like swelling and cytoplasm snouting), is a recently recognized subtype that can be of grade 1, 2, or 3. Since no death or distant metastasis was seen during follow-up, in this specific ECL-cell NET subtype, Ki67 index probably does not play a significant prognostic role [94].

#### Duodenum

Duodenal NETs include three different tumor types showing different morphological and clinical features: gastrinoma (functioning gastrin-producing NET), ampullary somatostatin-producing D-cell tumor, and non-functioning NET [95]. In addition to pure epithelial NETs, in the duodenum a fourth peculiar triphasic tumor composed of neuroendocrine, schwannian, and ganglion cells can be rarely observed. It was previously called gangliocytic paraganglioma, but it has renamed composite gangliocytoma/neuroma and neuroendocrine tumor (CoGNET) in the last 2022 WHO classification of neuroendocrine tumors arising in non-neuroendocrine organs [6]. As other digestive NETs, the duodenal ones are graded using proliferation indices, independently of the specific category. The prognostic role of Ki67 labelling index has been investigated and Ki67-based grading correlated with lymph node metastasis and, at univariate analysis, with disease-specific survival. However, Ki67 was not an independent prognosticator in G1 and G2 duodenal NETs at multivariate analysis [95]. When considering only ampullary NETs, grade proved to be a strong predictor of disease-specific survival together with patient age > 60 years, small-vessel invasion, pancreatic invasion, and distant metastasis at diagnosis [96]. G3 NETs were frequently associated with metastases suggesting that the clinical role of Ki67 expression becomes relevant when it reaches and exceeds 20% [97]. These findings suggest that Ki67 may be useful to identify duodenal NETs with increased risk of metastatic dissemination especially in G3 cases, although its impact on survival needs to be integrated with other parameters. The best prognostic approach appears to be multiparametric including Ki67 index, tumor size and duodenal wall infiltration [95, 96].

#### Ileum

Ileal NET is a peculiar tumor that, despite its small size, well-differentiated morphology and low Ki67 index (generally < 3%), in most cases deeply infiltrates the intestinal wall and is associated with loco-regional lymph node metastases at presentation [98, 99]. This feature suggests that Ki67 index is not a good predictor of metastatic potential in this specific site. However, Ki67 proliferative index has been found to correlate with prognosis using the cutoffs proposed by ENETS/WHO [89]. Interestingly, Panzuto et al. evaluated the risk of tumor progression and death for each increasing Ki67 unit that was 14% and 18%, respectively [100]. This approach appears biologically more correct than the use of fixed Ki67 cutoffs separating different categories, because Ki67 is a continuous variable.

#### Appendix

NETs of the appendix are generally small indolent tumors that, although frequently infiltrate through the appendiceal wall, rarely disseminate to local lymph nodes and almost never give distant metastases. The prognostic role of Ki67 labelling index has been explored in these tumors and some studies demonstrated that the distinction of grade 1 and grade 2 does not correlate with prognosis [101, 102], while others showed that Ki67 > 3% (grade 2), tumor size > 15.5 mm, and presence of lymphatic and vascular infiltration are associated with higher propensity to lymph node metastases [103]. However, the role and impact of lymph node metastases on patient’s prognosis still remains to be clarified.

#### Rectum

Rectal NETs are generally indolent tumors since in most of cases are diagnosed as small (< 10 mm) mucosal/submucosal lesions that can be easily resected during colonoscopy. In most of cases, rectal NETs are grade 1 (Ki67 < 3%) and these G1 NETs show a better survival than G2 NETs [104–106]. Although Ki67 correlated with prognosis at univariate analysis [105–107], it was not an independent prognosticator at the multivariate analysis [107] suggesting that a multiparametric approach evaluating Ki67 together with tumor size, lymphatic and vascular invasion, level of wall infiltration, and immunophenotype (L-cell versus EC-cell NET) may be the best tool to identify patients at higher risk of metastasis and tumor-related death.

#### Pancreas

The prognostic role of Ki67 proliferative index was investigated and demonstrated for the first time in pancreatic NETs (PanNETs) [3, 4], and since then, several other studies confirmed this observation [90]. For this reason, Ki67 index has become more and more important for the definition of PanNET behavior as demonstrated in multicentric studies including a large number of cases [108, 109]. The large body of evidence on the Ki67 prognostic role gained in PanNETs has encouraged and stimulated to explore it in other organs where, with some site- and tumor type-dependent peculiarities, it proved to play a relevant clinical role as observed in the pancreas. Consequently, Ki67 has been endorsed by WHO/IARC to be used for stratifying NETs in different prognostic categories [7]. The biological role of Ki67 in PanNETs has also been supported by a recent investigation demonstrating that gene expression profiles, mutational burden including *DAXX* and *ATRX* mutations, and LINE-1 methylation status correlate with Ki67 grade. In this study, the genetic similarities observed between G1 and G2 PanNETs suggest that only a few genes may play a role in the switch from indolent G1 to relatively aggressive G2 cases. Interestingly, the activation of a larger panel of genes was observed between G2 and G3 and, even more, between G3 and G1 PanNETs outlining the difference which exists between G1/G2 and G3 tumors [110]. All these findings suggest that Ki67 immunohistochemistry is mandatory in the work-up of PanNETs and that tumor grade represents the cornerstone for the prognostic evaluation of patients.

### Adrenal Cortical Neoplasms

Adrenal cortical neoplasms include adenoma and carcinoma that have been traditionally differentiated using specific classification systems based on morphological criteria not including Ki67 evaluation: the Weiss system [111], the reticulin algorithm [112], the Lin-Weiss-Bisceglia system for oncocytic neoplasms [113], and the AFIP system for pediatric neoplasms [114]. More recently, the Ki67 proliferative index, used as a continuous value and not fixed in established cut-offs, has been incorporated together with mitotic count and necrosis in a diagnostic system (the Helsinki scoring system, Table 5) [115], which has been validated in other series including a large number of cases [116]. Ki67 index > 5% is observed in most adult adrenocortical carcinomas [117–119], but this cutoff is not completely validated and Ki67 index seems to work better as a continuous variable as proposed in the Helsinki scoring system. For this reason, it has not been officially included in the new 2022 WHO classification as a diagnostic marker, although reporting Ki67 value is strongly recommended also for its prognostic and predictive relevance [6, 120]. Indeed, Ki67 index has been found to have a prognostic role in adrenal cortical carcinoma for which a Ki67-based prognostic score has been proposed to separate cancers into three groups with low (< 20%), intermediate (20–50%), and high (> 50%) Ki67 values [121]. More recently, the Ki67 index > 15% has been validated as prognostic cut-off in pediatric and adult cohorts being associated with higher risks of recurrence and/or poor outcome [118, 119, 122]. Interestingly, in pediatric patients, Ki67 < 15% was not associated with a clinical malignant behavior and consequently strongly recommended to be incorporated in the pathology report [118].

**Table 5:** The Helsinki scoring system for the diagnosis the adrenal cortical neoplasms. Modified from [115]

Morphological parameter	Specific score
Mitotic count: > 5 × 10 mm^2^	3
Presence of necrosis	5
Ki67 proliferative index (%)	Indicate the specific numeric value

Score: 0–8.5: benign, adrenal cortical adenoma; score > 8.5: malignant, adrenal cortical carcinoma; score > 17: adverse prognosis

**Fig. 7:**
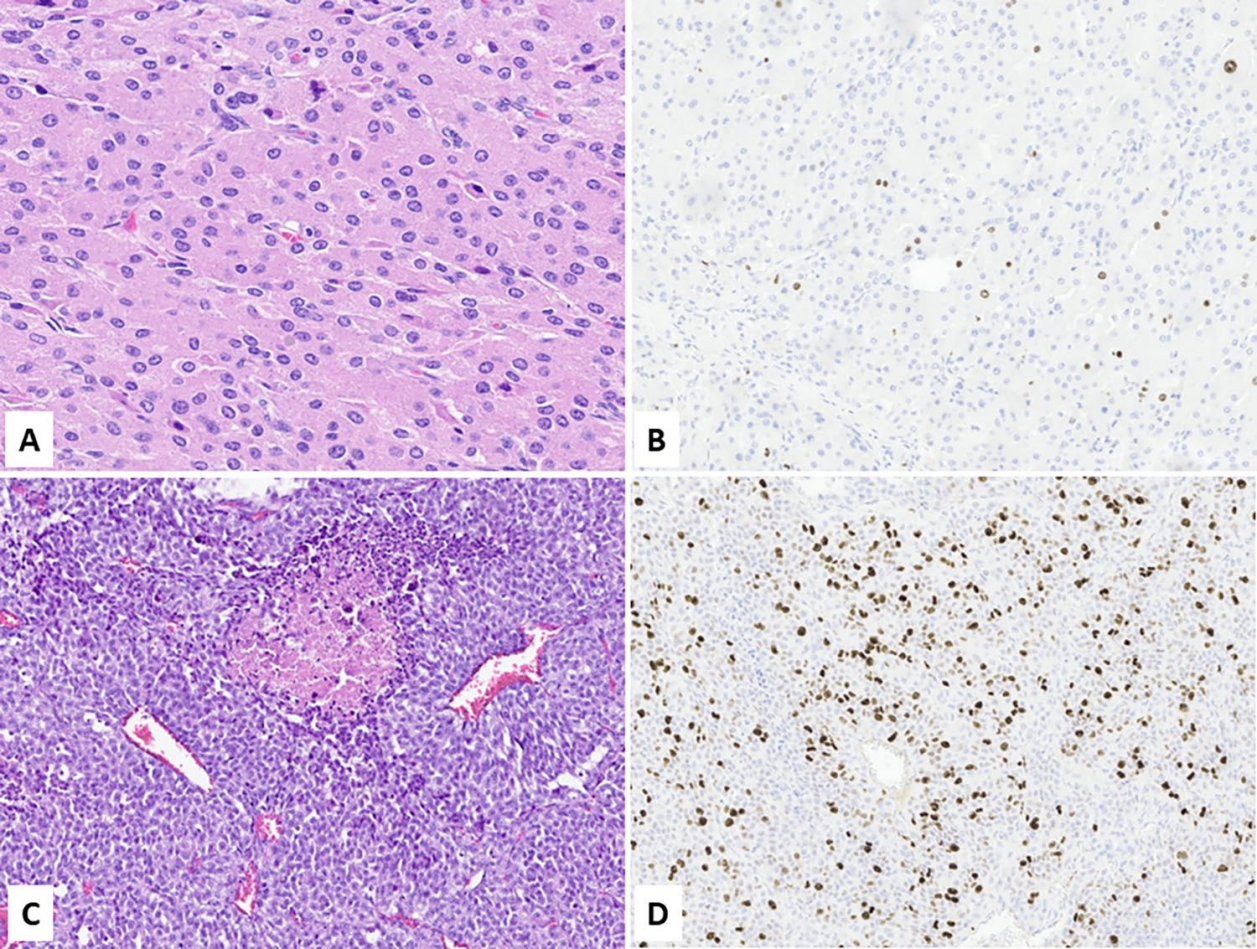
In **A** is presented an example of adrenal cortical carcinoma with Ki67 index < 10% (**B**), while in (**C)** an example of adrenal carcinoma with Ki67 > 10% (**D**). The Ki67 proliferative index plays a role in the selection of patients to treat with adjuvant mitotane therapy, indicated when Ki67 index is higher than 10%

The Ki67 proliferative index also plays a crucial role in the selection of patients to treat with adjuvant mitotane therapy, which, after radical surgery, is suggested for cancers with high risk of recurrence characterized by stage III, or R1 resection, or Ki67 index > 10% (Fig. 7) [123, 124].

In conclusion, although Ki67 proliferation index is not officially included as a diagnostic marker to differentiated adrenal cortical adenoma from carcinoma in the 5th WHO classification of endocrine and neuroendocrine tumors, it should be evaluated and considered a continuous numeric value to give a prognostic indication and to select patients to undergo mitotane therapy.

### Pheochromocytoma/Paraganglioma

**Fig. 8:**
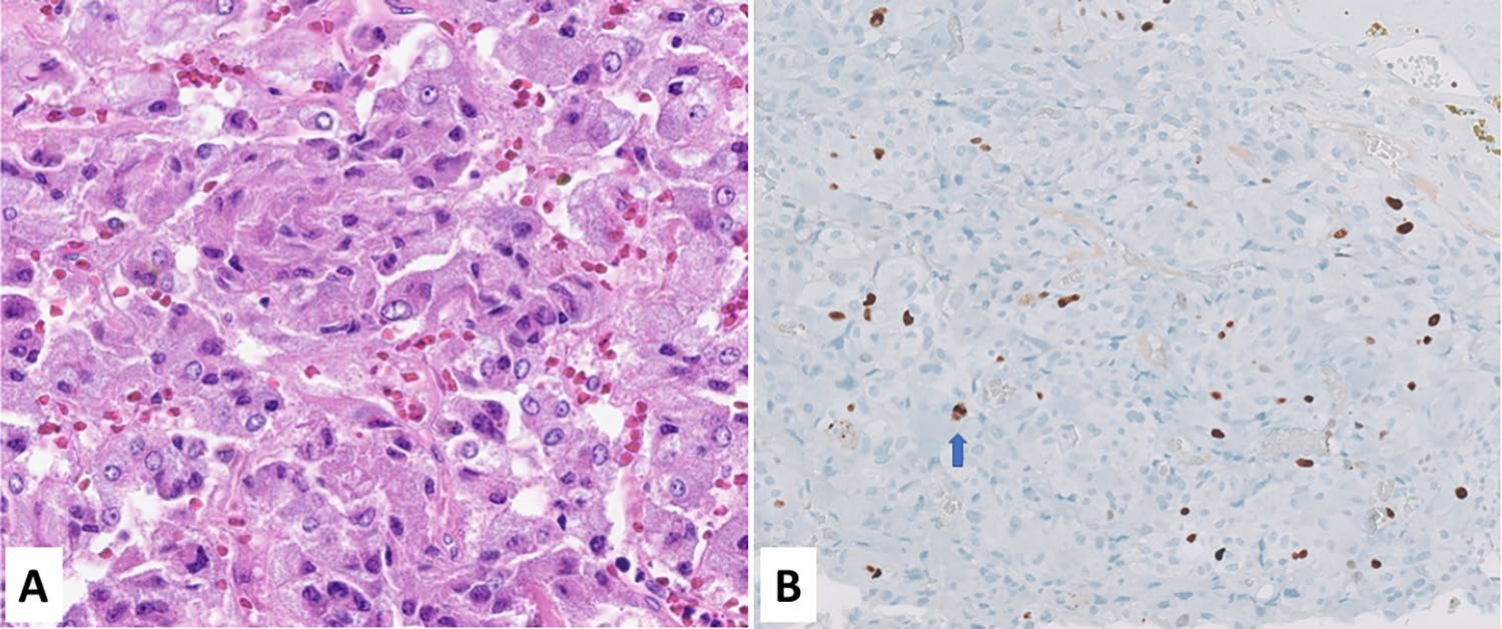
Pheochromocytoma (**A**) showing a Ki67 index of 3%, which has been proposed as the cutoff to separate patients in two different prognostic groups. In this case, a mitosis (arrow) is also observed in a Ki67 positive cell (**B**, courtesy of Prof. Silvia Uccella, Humanitas University, Milan, Italy)

The diagnosis of pheochromocytoma and paraganglioma is currently based on morphological and immunohistochemical criteria and, in this context, Ki67 immunolabelling does not have a diagnostic role. However, in the past, before the realization that these two tumor types are malignant, Ki67 expression was evaluated to explore its usefulness to differentiated “benign” from “malignant” neoplasms. In this context, Ki67 proliferative index has emerged as a reliable marker for identifying cases with high risk of metastatic dissemination and worse prognosis [125–127]. In particular, a Ki67 index > 3% was suggested to be prognostically relevant (Fig. 8), although it has not been confirmed in other recent studies [128].

As above mentioned, pheochromocytoma and paraganglioma are malignant neoplasms by definition. Once the diagnosis is morphologically and immunohistochemically performed, pathologists should search for parameters able to stratify patients in groups with different risk of metastasis and to this scope different systems have been proposed. The PASS score (pheochromocytoma of the Adrenal gland Scaled Score) was the first [129], and it was based on morphological criteria not including Ki67, which was successively integrated with growth pattern, cellularity, comedo-type necrosis, angioinvasion, and capsular invasion in the GAPP (Grading system for Adrenal Pheochromocytoma and Paraganglioma) system [130]. More recently, the COPPS score (Composite Pheochromocytoma/paraganglioma Prognostic Score), which is based on tumor size, the presence of necrosis and vascular invasion, and the loss of S100 and/or SDHB immunoreactivity but not including Ki67, has been proposed [131]. Although these systems have not proven to be sufficiently sensitive and specific to be endorsed for routine use, the 2022 WHO classification of pheochromocytoma and paraganglioma does not discourage their use [132]. As above mentioned, Ki67 is mainly considered as a prognostic marker, but it is worth noting that it may have a negative diagnostic role. Indeed, in pheochromocytomas and paragangliomas Ki67 proliferative index is generally less than 10% (Fig. 8) and a higher percentage should raise the suspicion that the tumor under examination is not a phaeochromocytoma or a paraganglioma [6].

In conclusion, Ki67 proliferative index only has a partial diagnostic role in the work-up of phaeochromocytoma and paraganglioma, but it can be useful, integrated with other prognostic marker, to identify those neoplasms at high risk of metastatic dissemination. For this reason, Ki67 immunohistochemistry is strongly recommended in the work-up of pheochromocytoma and paraganglioma [133].

### Urogenital Neuroendocrine Tumors

Neuroendocrine neoplasms of the urinary tract and male genital system have been traditionally defined and classified in the old literature using different terms. Taking into account the WHO/IARC proposition of a common classification framework unifying the nomenclature of NENs arising in different organs [7], the 5th edition of WHO classification of urinary and male genital tumors dedicates a specific chapter to NENs and classifies them into NET and NEC [134]. The diagnosis is based on morphology and immunohistochemical profile and Ki67 does not play a major role in this setting. However, Ki67 may play a prognostic role but, due to rarity of these neoplasm, it needs to be definitively determined. Promising findings suggest that a Ki67 index > 3% is associated with worse behavior [135, 136].

In the last WHO classification of female genital tumors (5th edition) NENs are discussed in a specific chapter and separated into NET, NEC, and MiNEN following the WHO/IARC scheme [7]. The diagnostic criteria are based on morphology and tumor grade is based on mitotic count and on the presence/absence of necrosis, with grade 1 NET showing no necrosis and < 5 mitoses per 2mm^2^, and grade 2 NET with 5–10 mitoses per 2mm^2^ and necrosis. Strangely and not in line with the WHO/IARC recommendation, Ki67 is not included as a parameter to be considered for grading NETs [137], and this may represent a diagnostic weak point for the identification of the rare G3 NETs that, although rare, can also be observed in this system [138]. Although in some studies Ki67 has been investigated [139, 140] its real prognostic role in NET of the female genital tract still remains to be validated.

### NECs

NECs are grade 3 cancers associated with dismal prognosis. Their diagnosis is based on the specific histological features and is supported by the demonstration of a neuroendocrine phenotype that includes the expression of synaptophysin, INSM1, and chromogranin, the latter being focally expressed or even negative in a not negligible number of cases. Ki67 index is by definition > 20% (digestive system) or > 30% (lung), but not rarely it reaches 70–80% or more helping in the differential diagnosis with G3-NETs that have a Ki67 index > 20% but which rarely exceeds 50–60%. The differential diagnosis between G3-NET and NEC in difficult cases can be made by integrating the Ki67 proliferative index with p53 and Rb immunohistochemistry, since most NECs show *TP53* and *RB* mutations and consequent p53 and Rb aberrant immunohistochemical expression. The prognostic role of Ki67 in NECs has been evaluated. The first study demonstrating that the cutoff of Ki67 index of 55% was able to separate G3 NENs in two different prognostic groups, which also differently responded to platinum-based chemotherapy, was published in 2013 [141]. Although this investigation represented the start point to better understand the heterogeneity of G3-NENs, it suffered from the lack of morphological revision of enrolled cases. However, this cutoff has been demonstrated to have a prognostic role in well-characterized series of NECs where the rare NECs with Ki67 < 55% behave in a better fashion than those with Ki67 > 55% [142]. For this reason, Ki67 immunohistochemistry is suggested also when diagnosing NECs.

The role of Ki67 index in Merkel cell carcinoma (MCC) has been investigated as well. The Ki67 cutoff value of 55% was found to be able to separate two distinct prognostic groups with that showing Ki67 > 55% associated with a worse outcome, which also depends on stage IV, lack of Merkel cell Polyoma Virus (MCPyV), and p63 expression. However, at multivariate analysis, survival resulted independently influenced only by p63 expression and tumor stage [143].

## Technical Issues and Practical Suggestions for the Evaluation of Ki67 Proliferative Index

**Fig. 9:**
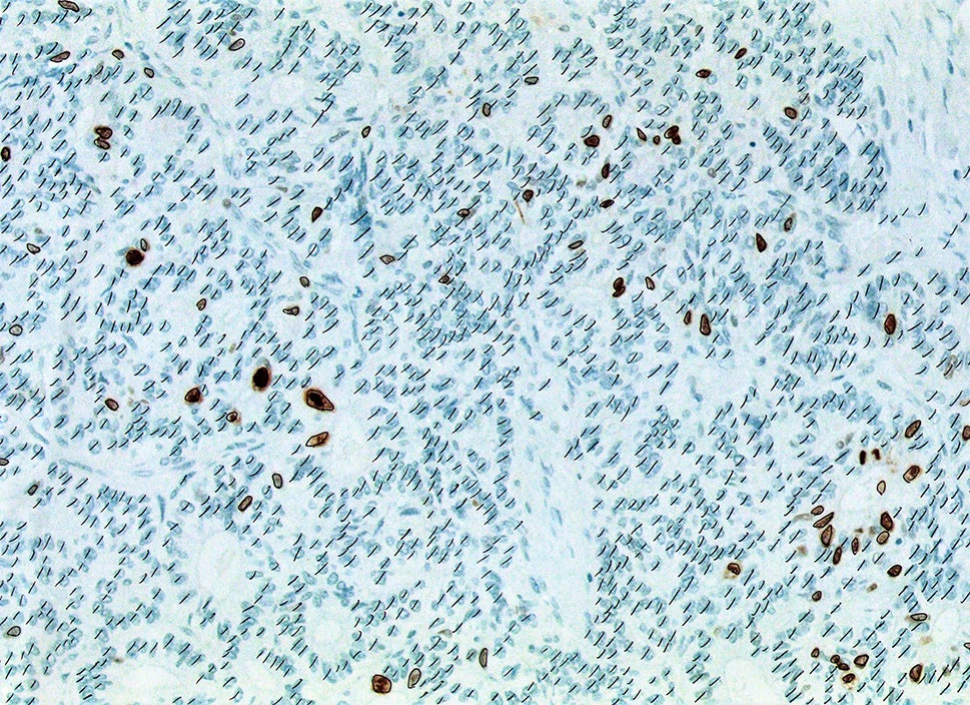
Example of Ki67 index evaluated by manually counting unlabeled and labeled nuclei on a camera-captured, printed image. In this picture, 81 Ki67-labeled cells were counted and then divided by a total of 1581 cells, resulting in a Ki67 index of 5.1% (republished with permission of Springer from the article: Klöppel et al. [90] Ki67 labeling index: assessment and prognostic role in gastroenteropancreatic neuroendocrine neoplasms. Virchows Arch 472:341–349)

**Fig. 10:**
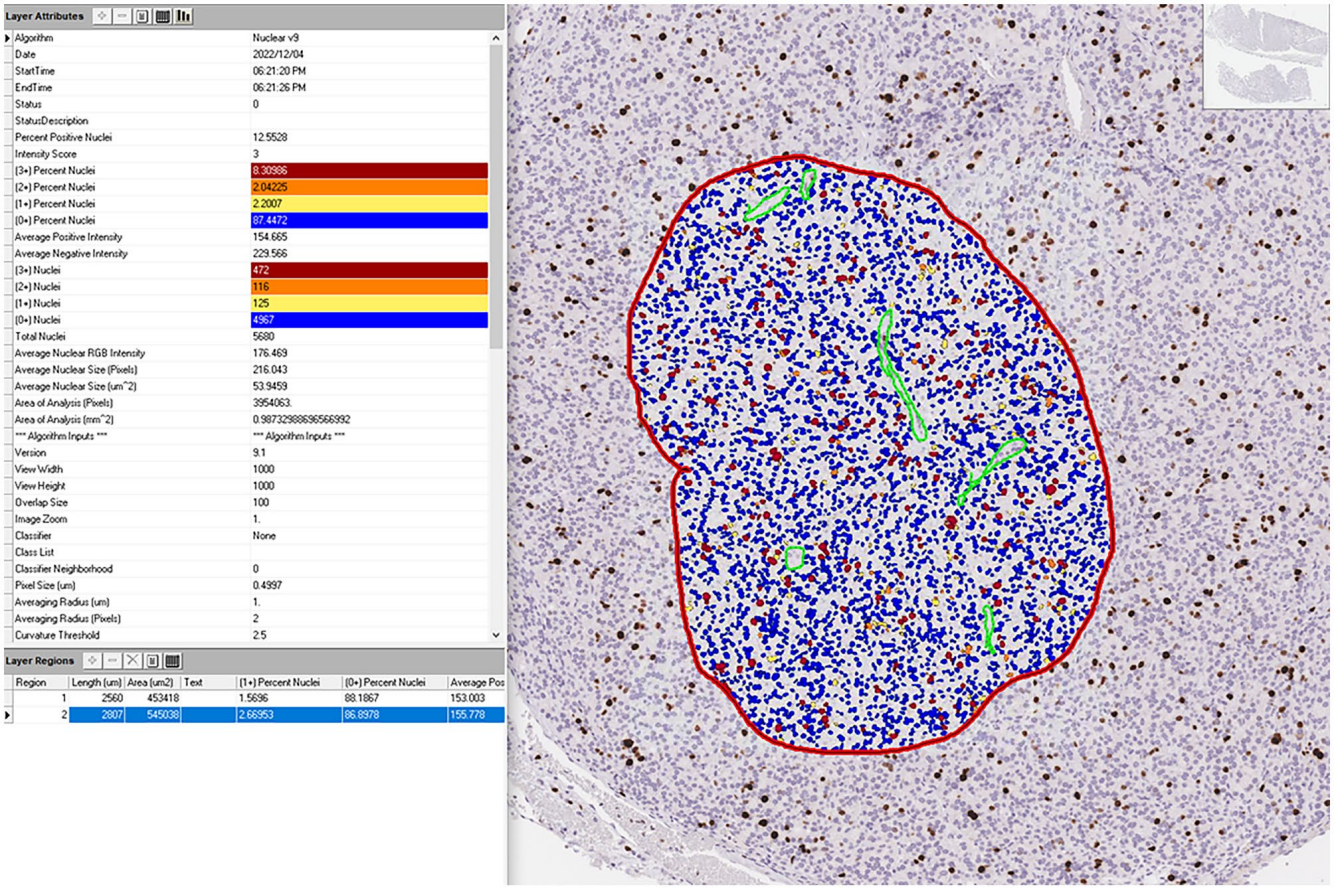
Example of automatic Ki67 count within a poorly differentiated thyroid carcinoma (courtesy of prof. Ozgur Mete, University Health Network, University of Toronto, Toronto, ON, Canada)

Ki67 is expressed in proliferating cells, but with a variable nuclear distribution among the different phases of the cell cycle [144]. For this reason, all immunoreactive nuclei, independently of the intranuclear distribution or intensity of the staining, should be scored [145]. The Ki67 index is expressed as the percentage of Ki67 immunoreactive cells in at least 500 tumor cells counted in the highest labeled area (“hot spot”). The best method to perform this count has been matter of debate in the last years and different systems have been proposed: eyeballing evaluation, automatic computer-assisted count, manual count at the microscope, and manual count on camera-capture printed images. Among them, manual count of Ki67 positive nuclei in camera-captured printed images has appeared to be the most reliable procedure (Fig. 9) [146]. It takes an average time between 10 and 15 min (longer than the other methods) but shows good reproducibility and can be used by most pathologists, since a camera-equipped microscope is available in most laboratories. Automated counting seems to have comparable accuracy (Fig. 10), but it needs a specific software not available in all laboratories [147].

Although immunohistochemical techniques have been improved and standardized in the last years with the introduction of automated staining machines, the Ki67 immunostaining is a delicate procedure influenced by several factors including tissue processing and fixation, the use of different reagents and pretreatments [148], which result in well documented interlaboratory variability [149]. For this reason, each laboratory should optimize the procedure to warrant a reproducible result.

## Future Perspectives

Published papers on Ki67 expression in endocrine and neuroendocrine neoplasms have generally demonstrated that the Ki67 proliferative index plays a prognostic role, either alone (i.e., in the pancreas) or in combination with other morphological parameters including tumor subtype (i.e., in the stomach and pituitary), local invasion (i.e., in the pituitary), mitotic count and necrosis (i.e., in the lung) or necrosis alone (in the thyroid). However, the value of Ki67 index is not always the same and some specific differences in its prognostic power depend on tumor type and its site of origin.

It is worth noting that in term of biological meaning the current application of Ki67 proliferative index presents a conceptual limit. Ki67 has been considered as a static parameter, a choice due to the need to have practical values useful to stratify patients in prognostic groups easy to be applied in routine diagnostic work. However, Ki67 index should be considered as a continue variable as done in the Helsinki score for adrenal cortical neoplasms [115] and this approach may better reflect its biological meaning. It is clear that grouping NENs using strict cutoff values, although easy, put together neoplasm with different biological aggressiveness as demonstrated by digestive G2-NET group where cases with Ki67 index of 3% are grouped with cases showing a higher Ki67 index (i.e., 19%). In this context, the use of a Ki67-related biological risk of tumor recurrence or tumor-related death may appear more accurate. In some studies, the Ki67-related biological risk has been evaluated [100, 150]. In lung ACTH-secreting NETs a 1.41 increased risk of recurrence has been noted for each 1% of Ki67 index [150]. In ileal NETs, a 18% increased risk of death for each increasing unit of Ki67 index has been found [100]. This approach seems relevant and further studies are needed to better evaluate its role in different NET types.

The predictive role of Ki67 for therapeutic purpose needs to be better explored in both neuroendocrine and endocrine neoplasms and now is only well established to select patients with adrenal cortical carcinomas who can be treated with mitotane.

## Data Availability

Published literature.
